# The function of communities in protein interaction networks at multiple scales

**DOI:** 10.1186/1752-0509-4-100

**Published:** 2010-07-22

**Authors:** Anna CF Lewis, Nick S Jones, Mason A Porter, Deane M Charlotte

**Affiliations:** 1 Department of Statistics, University of Oxford, Oxford, UK; 2 Department of Physics, University of Oxford, Oxford, UK; 3 CABDyN Complexity Centre, University of Oxford, Oxford, UK; 4 Department of Biochemistry, University of Oxford, Oxford, UK; 5 Oxford Centre for Integrative Systems Biology, University of Oxford, Oxford, UK; 6 Oxford Centre for Industrial and Applied Mathematics, Mathematical Institute, University of Oxford, Oxford, UK

## Abstract

**Background:**

If biology is modular then clusters, or communities, of proteins derived using only protein interaction network structure should define protein modules with similar biological roles. We investigate the link between biological modules and network communities in yeast and its relationship to the scale at which we probe the network.

**Results:**

Our results demonstrate that the functional homogeneity of communities depends on the scale selected, and that almost all proteins lie in a functionally homogeneous community at some scale. We judge functional homogeneity using a novel test and three independent characterizations of protein function, and find a high degree of overlap between these measures. We show that a high mean clustering coefficient of a community can be used to identify those that are functionally homogeneous. By tracing the community membership of a protein through multiple scales we demonstrate how our approach could be useful to biologists focusing on a particular protein.

**Conclusions:**

We show that there is no one scale of interest in the community structure of the yeast protein interaction network, but we can identify the range of resolution parameters that yield the most functionally coherent communities, and predict which communities are most likely to be functionally homogeneous.

## Background

Large protein-protein interaction data sets [1-3] and functional information about many proteins are increasingly available. This allows one to investigate the patterns in protein-protein interactions that enable proteins to act concertedly to carry out their functions. In particular, considerable recent attention has been given to the modularity of the cell's functional organisation [4-6]. A module is often thought of as a group of components that carry out a functional task fairly independently from the rest of the system. It is thought that such modules yield robust and adaptable systems [7]. There is also much suggestive evidence that modules within the cell are themselves the building blocks of a higher level of structural organisation (e.g. [8-10]).

Within the networks literature a great many algorithms have been proposed that locate dense regions in a network, often called communities (reviewed in [11,12]). A community is loosely defined as a group of nodes that are more closely associated with themselves than with the rest of the network. Such communities are potentially good candidates for functional modules, and many studies report running one of the myriad algorithms for detecting community structure on protein interaction networks [13-19]. Having located communities, such studies then attempt to assess their functional homogeneity by searching for terms in a structured vocabulary --usually the Gene Ontology (GO, [20]) or Munich Information Centre for Protein Sequences categories (MIPS, [21])--that are significantly over-represented within communities. If such terms exist, the identified communities are said to be 'enriched' for biological function. In many studies such enriched communities are found, and hence are plausible candidates for biological modules.

Recently there has been an acknowledgement that many community detection algorithms - in particular all those that rely on optimising the quality function known as modularity - impose an artificial *resolution limit *on the communities detected [22]. Such algorithms return communities found at one particular resolution - i.e. at one particular scale within the network - whereas there are many scales of potential functional relevance within the protein interaction network. For example, one might expect to find smaller communities embedded inside progressively larger ones [11]. There are now algorithms available that include a 'resolution parameter', which allow one to uncover structure at many different resolutions [23-29]. However, no study to our knowledge has systematically applied such an algorithm and analysed the results across different resolutions in protein interaction networks (one study reports testing more than one value of a parameter akin to the resolution on a protein interaction network, in order to select an optimal value for their purposes [30]).

In this study, we probe the functional relevance of communities at multiple resolutions (scales) in the yeast protein interaction network, for two main biological reasons. First, considering the whole proteome, it is possible to view how the network breaks into communities (hierarchically or otherwise), and to investigate whether some scales of organisation are of more relevance than others biologically. Second, the relationship of multi-scale community structure to a particular protein is of interest: it is possible to see which other proteins co-occur with it at different resolutions - perhaps it co-occurs robustly with a small group of proteins at high resolution but also with a larger set of proteins at a lower resolution. Both groups are of potential interest in understanding what role the protein plays. This is particularly pertinent for poorly annotated proteins, as their patterns of potential function can be revealed through clustering into communities [31].

Although it is already thought that communities have some relationship to functional modules, here we expand on previous work to assess the functional relevance of communities in four main ways.

First, assessing functional relevance by counting over-represented terms amongst a group of proteins is not a sufficiently stringent test of functional relevance when the group of proteins in question is a community. This is because two proteins that interact are functionally more similar than a randomly chosen pair of proteins, so one must control for the number of interactions when assessing the biological relevance of a community (which will necessarily include more interacting pairs than a randomly selected group of proteins). We therefore control for the number of interacting proteins found in a community.

Second, instead of assessing functional homogeneity on a term by term basis we use all the annotations available within a given ontology.

Third, GO and MIPS are subjective by their nature, both in the definition of the sets of terms themselves and in the process of annotation of terms to proteins. Due to their role in a particular process, a protein might well be both annotated more fully and have a higher probability of having had protein interaction experiments performed on it. Therefore, in addition to using GO and MIPS as protein functional characterizations, we use a single high-throughput experiment on the growth rates of gene knock-out strains under various conditions (using data from [32]).

Fourth, protein interactions are of two fundamentally different types. The Molecular Interactions ontology [33] recognises two distinct types of interactions: physical associations (henceforth denoted *P*) and associations (henceforth denoted *A*). The main experimental type for the former are yeast-two-hybrid screens (e.g. [34]). The main type of experiment to fall under the latter are based on tandem affinity purification (TAP, e.g. [35]). These interaction types are known to have very different properties [1,36]. Additionally, the networks constructed using these two types of interactions have quite different global properties (see Table 1). We thus investigate the two networks, based on type *A *and type *P *interactions, independently.

**Table 1:** Network statistics of the *A *and *P *networks

Network	*A*	*P*
Number of nodes	4980	5669
Number of edges (of which self edges)	48,330 (868)	33,321 (941)
Mean degree	19.1	11.5
Mean clustering coefficient	0.22	0.10

We identify communities at multiple resolutions in these two fundamentally different interaction networks. We then use novel tests to determine the communities' functional homogeneity using three different characterisations of function. As the functional knowledge of proteins is far from complete (even for well characterised organisms such as yeast), we also search for topological properties of communities that are correlated with functional homogeneity.

In our study we find many functionally homogeneous communities at multiple network resolutions. Almost all proteins are in functionally homogeneous communities at some resolution (4652 of 4980 proteins in the *A *network, and 5647 of 5669 proteins in the *P *network). The resolution that places most proteins in functionally homogeneous communities is beyond the 'resolution limit', or standard resolution, discussed above. At this maximum, 3071 out of 4980 proteins are in functionally homogeneous communities according to our GO similarity measure in the *A *network. Communities at this resolution have mean size 73, compared to mean size 293 at the standard resolution. We find similar numbers for the *P *network. Additionally, we find a high degree of overlap between communities judged functionally homogeneous using three separate quantifications of functional similarity. Through a further characterization of the communities using 26 topological properties, we identify the mean clustering coefficient of a community as a good predictor of functional homogeneity, with a true positive rate of 70% achievable with a false positive rate of 30%. In addition to these proteome-scale results, we demonstrate via examples how this approach can be used to predict groups of proteins likely involved in similar processes to a particular protein of interest.

## Methods

### Protein-Protein Interaction Datasets

Here we use the BioGrid (http://www.thebiogrid.org, downloaded January 2010, [37]), IntAct (http://www.ebi.ac.uk/intact, downloaded January 2010, [38]) and Mint databases (http://mint.bio.uniroma2.it/mint, downloaded January 2010, [39]) to assemble our protein interaction networks. We use only interactions between proteins that have an SGD identification (Saccharomyces Genome Database, http://www.yeastgenome.org, [40]).

We divide interactions on the basis of their type (*A *or *P*) and hence assemble the two networks. The IntAct database [38] gives interaction types from the Molecular Interaction ontology [33] directly. It contains 23632 interactions of type *A *and 26611 of type *P*. The Mint database [39] uses the Molecular Interaction interaction detection type ontology, the broad categories of which are biophysical, biochemical, and protein complementation assay. The biochemical techniques give evidence of association (type *A *interactions), and the biophysical and protein complementation assays give evidence of physical interactions (type *P*). Using this division, there are 13347 *A *type interactions and 10407 *P *type interactions. The BioGrid database [37] uses its own evidence types. Those giving evidence of *P *type interactions are reconstituted complex, PCA, Co-crystal structure and yeast-two-hybrid. Those giving evidence of type *A *interactions are affinity capture, biochemical activity, co-fractionation, co-purification and Far Western. (Details of these experimental types can be found on the BioGrid website, http://www.thebiogrid.org). There are 35716 *A *type interactions and 13142 *P *type interactions overall. Of the potential 6607 proteins in the yeast proteome http://www.yeastgenome.org, there are 5002 proteins connected by *A *type interactions, and 5692 connected by *P *type interactions. Here we only study the largest connected component of these networks, leaving 4980 proteins in the *A *network and 5669 in the *P *network. Some summary statistics for the two amalgamated networks are shown in Table 1. The *A *network is denser, and has higher clustering. There are 5947 interactions in common between the *A *and the *P *networks.

### Potts community detection

We apply the Potts method [23]. It partitions the proteins into communities at many different values of a resolution parameter, thus finding communities at different scales within the network. The method seeks a partition of nodes into communities that minimises a quality function ('energy'):(1)

where *s_i _*is the community of node *i, δ *is the Kronecker delta, *λ *is the resolution parameter, and the interaction matrix *J*_*ij *_(*λ*) gives an indication of how much more connected two nodes are than one would expect at random (i.e., in comparison to some null hypothesis). The energy *H *is thus given by a sum of elements of *J *for which the two nodes are in the same community. Optimising *H *is known to be an NP-hard problem [41,42], so one must use a computational heuristic. Here we use the greedy algorithm discussed in [43] and freely available http://www.lambiotte.be/codes.html, which performs well against various benchmark tests [44]. As pointed out by Good et al [45], one must be cautious in interpreting results obtained from detecting communities by optimizing modularity or similar quality functions, as there is a degeneracy of partitions with almost optimal *H*. As a consequence different optimisation techniques can find very different optima.

The interaction matrix *J *has elements(2)

where the matrix *B *with elements *B*_*ij *_is the adjacency matrix. In this case *B*_*ij *_= 1 if proteins *i *and *j *interact, and *B*_*ij *_= 0 otherwise. The matrix *R *with elements *R*_*ij *_defines a null model, against which we are comparing the network of interest. Here we choose the standard *Newman-Girvan *null model [46], which has the property that it preserves the expected node degree sequence. That is,(3)

where *k*_*i *_= ∑ _*j *_*B*_*ij *_is the degree of node *i*, and *W =∑ *_*ij *_*B*_*ij*_*/2 *is the number of edges in the network. When *λ *= 1, *H *is the standard Newman-Girvan modularity quality function, upon which many community detection algorithms are based [11,46]. We hence refer to this value of the resolution parameter as the standard resolution. Values of *λ *> 1 probe the network at resolutions above the resolution limit.

We investigate partitions of the network in the range 0.1 is ≤ *λ *≤ 1000, and sample at intervals of 0.01 on a logarithmic scale (we hence report results for -1 ≤ log(*λ*) ≤ 3). At *λ *= 0, all nodes in our set will be assigned to the same community. As we increase *λ*, communities split and become smaller. If we allow *λ *to increase until all of the entries in *J*_*ij *_are negative, then each node will be assigned to its own community.

### Convention for identifying communities at different partitions

To relate the partition at one value of the resolution parameter *λ *to that at another (which we use here for visualisation), we require a convention for labelling communities. Here we use a method based on the overlap of shared nodes [47]. A convention based on links rather than nodes gives nearly identical results. Let the communities in the first partition (which here is that at the highest resolution) be labeled *K*_*1*_, ..,*K*_*s*_, and those in the next partition be labelled *L*_*1*_*, ...,L*_*t*_. Then for each pair of communities, {*K_i_, L_j_*}, we have(4)

where |*B*| denotes the cardinality (number of elements) of the set *B*. Starting with the largest value of *W*_*ij *_, we relabel community *i *as community *j*. Relabelling proceeds with the next largest *W*_*ij *_, as long as community *i *is not yet relabelled, until all communities have been relabelled. If *s *>*t*, we introduce a new label.

### Pairwise measures of functional similarity

It is impossible to uniquely quantify similarity in biological function. Here we rely primarily on the GO http://www.geneontology.org, which provides the most comprehensive available database of functional annotations. We use the Biological Process sub-ontology annotations to yeast, which are maintained by the SGD consortium [40]. Terms are related to each other through a directed acyclic graph (DAG). Proteins are annotated with the most specific terms that are known about them. It is then possible to add to this set their parent terms by following the structure of the DAG, up to the root node. Well-characterised proteins are those annotated with terms far from the root node. Of the 6346 yeast proteins in the GO annotation set, 5347 have biological process annotations (excluding the root node). We carried out the same tests using the Molecular Function and Cellular Component sub-ontologies, which gave similar results.

We also use MIPS terms (http://www.helmholtz-muenchen.de/en/ibis, [21]), which are a useful double check on our results from GO, and have the added advantage that the terms are all found at the same level within the hierarchy of terms. Here we only use the top level of the MIPS hierarchy.

Following [48], we quantify the functional similarity between two proteins *i *and *j *by finding the set of GO terms annotated to both proteins and counting the total number of proteins, *n*_*ij *_, that share that set of terms. We then define a similarity measure between proteins *i *and *j *as(5)

where *N *is the total number of proteins. If both proteins are annotated with a set of terms that few proteins share, then they will be judged as functionally similar under this measure. Unlike many other measures, *G*_*ij *_does not penalise proteins for lack of annotation when judging their similarity. This is desirable, as we know that the GO annotations (even for the well-characterised *S. cerevisiae*) are far from complete. The quantity *M*_*ij *_is similarly defined through Equation 5 for the MIPS annotations.

The benefit of using a pairwise similarity measure that takes into account the full set of functional information available, rather than examining enrichment of function on a term by term basis, is that the measure has the potential to capture more general functional similarities between a pair of proteins.

We also define a similarity between two proteins from a single high-throughput experiment via the growth rates of knock-out strains under a range of different conditions. Using the data in [32], we define *C*_*ij *_, the correlation in growth rates of the strain with gene *i *knocked out to the strain with gene *j *knocked out under 418 different conditions:(6)

where the elements of the vector *L_i _*are(7)

the parameter  is the mean growth rate of strain *i *under different control conditions, and  is the growth rate under one of the 418 treatment conditions. We use the results from the homozygous strains. Because many gene deletions are lethal, there is only data available for 3625 proteins, of which 3184 are in the *A *network and 3422 are in the *P *network.

### Assessment of a community's functional homogeneity

As mentioned previously, a fair test of the functional homogeneity of a community must take into account the fact that a pair of proteins that interact will be more similar than a randomly chosen pair. Standard enrichment tests do not take this into account, as they compare enrichment in a group of proteins, in this case a community, to what one would expect to attain from a randomly chosen set of proteins [49]. A community necessarily contains many more interacting pairs than a randomly chosen set. We thus compare the pairwise functional similarities of all interacting pairs of proteins in a community to the same measure for all interacting pairs in the network, thereby controlling for the number of interacting pairs.

To capture the pairwise similarity between two proteins that interact {*ij*}, we use *z*-scores:(8)

Where *S *stands for one of our three similarity measures (based on GO, *G*, MIPS, *M*, or correlated growth rates, *C*), *μ *is the mean and *α *the standard deviation of all of the elements of *S *for which proteins *i *and *j *interact in the network of interest (*A *or *P*).

A desirable quality for our test of functional homogeneity is the ability to compare communities found at different resolutions in an even handed manner. It is inherent in the nature of a statistical test that the significance of the test statistic under consideration (for example, the difference between the sample mean and the population mean) depends on the sample size: if one has a larger sample size, one can judge smaller differences to be 'significant'. To determine the aggregate *z*-score, *z*_agg_, for the mean of a set of individual *z*-scores, *z*_ind_, one calculates , where *N *is the number of *z*_inds _and *μ*(*z*_ind_) is their mean [50]. So, given a *μ*(*z*_ind_), a larger and hence more significant *z*_agg _is achieved for a larger sample size (i.e. larger *N*). In order to separate out the effects of the number of interactors in the community from functional homogeneity, we thus choose to base assessment of functional homogeneity on the *μ*(*z*_ind_), in our case *μ*(*z*_{*ij*}_) (*z*_{*ij*} _is defined in Equation 8). We judge as 'significant' all those communities that have *μ*(*z*_{*ij*}_) above 0.3, and call such communities "functionally homogeneous". We stress that this is not strictly an assessment of statistical significance, as we are choosing to ignore sample size. The value of 0.3 would be judged to be significant at the 0.05 significance level for any community with 30 or more interacting pairs.

### Classification of protein types

We focus on a small but broad set of protein types, which are the GO biological process terms within the yeast GO slim [51] that are annotated to at least 200 yeast proteins. They are (numbers of proteins in brackets): 1. DNA metabolic process (357); 2. protein modification process (465); 3. transport (859); 4. response to stress (458); 5. membrane organization (208); 6. RNA metabolic process (715); 7. vesicle-mediated transport (280); 8. response to chemical stimulus (298); 9. cellular lipid metabolic process (204); 10. cellular carbohydrate metabolic process (220) and 11. chromosome organization (338).

### Topological properties that correlate well with functional homogeneity

We investigate 26 topological properties of the identified communities and assess whether any of these can be used to identify functionally homogeneous communities. Examples include mean clustering coefficient, betweenness measures, and network diameter. Any topological properties that correlate well with functional homogeneity can then be used to predict functionally homogeneous communities. We use each topological property as a classifier by predicting communities as functionally homogeneous when the value of that property is above a threshold, which we vary to construct a Receiver Operating Characteristic (ROC) curve. An ROC curve plots the number of communities correctly predicted as functionally homogeneous versus the number falsely predicted [52]. We calculate the area under the ROC curve (AUC) for each metric at each value of *λ*, and report the mean of this quantity over resolutions between 0 ≤ log(*λ*) ≤ 3 (we exclude -1 ≤ log(*λ*) < 0, as the results are very noisy due to the small number of communities present). An AUC of 0.5 would be expected from a random classifier. AUCs of greater than 0.5 imply that higher values of the metric are predictive of functional homogeneity. AUCs of less than 0.5 imply predictive power if *below *a threshold of that particular property was used (i.e. that the property and functional homogeneity are negatively correlated).

## Results and Discussion

### Pairwise properties of proteins

Community structure, if of any biological relevance, should uncover patterns that are more than the sum of effects from pairs of interacting proteins. In Table 2 we show the pairwise similarity of proteins in each network under our three different measures of functional similarity (based on GO, MIPS, and correlated growth rates; see Methods). The similarity of pairs known to interact with either *A *or *P *type interactions is much higher than a randomly chosen pair of proteins under all three measures. This both helps motivate the investigation of the connection between functional similarity of proteins and the topology of the network, and demonstrates the necessity of taking into account pairwise properties when assessing any additional information that one can gain by studying communities.

**Table 2:** Pairwise similarities of proteins in the *A *and *P *networks under the three different similarity measures, *G, C*, and *M*

	*A*		*P*	
	All pairs	Interacting pairs	All pairs	Interacting pairs
*G*	0.04	0.14	0.04	0.12
*C*	0.19	0.35	0.18	0.33
*M*	0.22	0.28	0.22	0.27

### Communities

**Figure 1:**
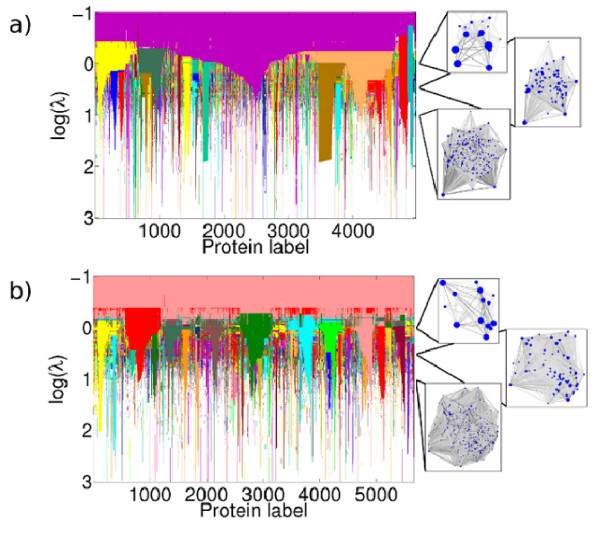
**Communities identified in the *A *and *P *Networks**. Communities identified in the yeast protein interaction network for interactions of a) type *A *and b) type *P*. When the resolution parameter *λ* is very small, all nodes are assigned to the same community (which is analogous to viewing the network at a great distance). As *λ* is increased (viewing the network at progressively closer distances), more structure is revealed. The figures on the right hand side show visualisations of the networks' partition into communities at three different values of *λ*. Each circle represents a community, with size proportional to the number of proteins in that community, positioned at the mean position of its constituent nodes. (These positions were determined via a standard force directed network layout algorithm [57].) The shade of the connecting lines is proportional to the number of links between two communities. The main figure shows the communities that we find as we vary the resolution. We identify communities as the same through changing resolution parameter, and hence colour them the same, according to a convention described in the Methods (only communities of size 50 or more are shown). Note that the ordering of proteins is not the same in the two figures.

Figure 1 shows the communities that we find in the *A *and *P *yeast networks as the resolution parameter *λ *is varied. As *λ *increases, more and smaller communities are found (see Table 3). At *λ *= 1 (i.e. log(*λ*) = 0), which corresponds to standard Newman-Girvan modularity [46], most communities contain a few hundred proteins. By log(*λ*) = 3 however, almost all proteins are in communities of size three or smaller. As shown in Figure 1, some sets of nodes are classified in the same community through large changes in the resolution parameter and hence represent particularly inter-connected parts of the network. Figure 1 can be contrasted with Figures S1 in Additional File 1, which are similar calculations on a random network and a network designed to possess strong communities. In the former, not much structure is present, in the latter, there are very distinct blocks.

**Table 3:** Mean size of communities in the *A *and *P *networks

log(*λ*)	mean size of communities	
	*A*	*P*
-0.5	681	2834
0	293	405
0.5	73	79
1	22	26
1.5	11	10
2	6	6
2.5	5	5
3	4	4

**Figure 2:**
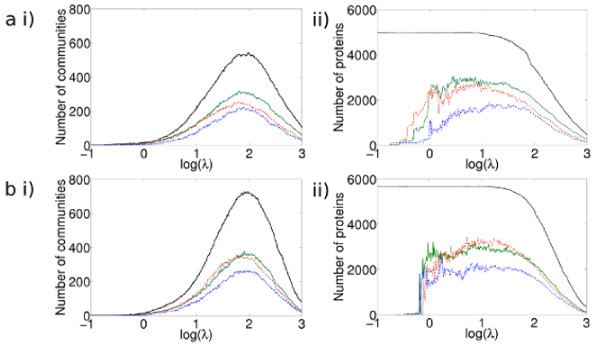
**For a) the *A *network b) the *P *network, i) the number of communities of size four or more and ii) the number of proteins in such communities and the fraction of these that are judged functionally homogeneous**. i) The number of communities with changing resolution parameter (solid black curve) ii) The number of proteins *p *in communities of size four or more (solid black curve). Also shown are the numbers of communities/proteins in such communities judged to be functionally homogeneous according to the GO similarity measure (green curves), the MIPS measure (dot-dashed blue curves) and the correlated growth similarity measure (dashed red curves). At values of log(*λ*) ≤ 0.5, relatively few proteins are in communities judged to be functionally homogeneous. The curves are similar for both networks, and they show a similar proportion of proteins in functionally homogeneous communities. One difference is that there are more proteins in functionally homogeneous communities at a lower value of log(*λ*) for the *P *network.

The black lines in Figure 2 illustrate for a) the *A *network and b) the *P *network, i) the number of communities of size four or more as the resolution changes, and ii) how many proteins are in those communities.

The two networks, *A *and *P*, contain very different types of interactions, and they can therefore be used to identify different aspects of the cell's functional organisation. The *A *network is also much denser than the *P *network. *A *interactions would therefore dominate the clustering into communities, thereby making it very hard to pick out any structures given by *P *type interactions (as occurs in [53]). When considering a particular protein or set of proteins, comparisons between communities found in the *A *and *P *networks can be made, see the Examples section. Global comparisons between the partitions of the *A *and *P *networks at a particular resolution are not necessarily meaningful as, for example, the size of communities depends both on the size and other properties of the network.

Data files containing the *A *and *P *networks and the community membership of proteins at multiple resolutions are available at http://www.stats.ox.ac.uk/research/proteins/resources.

### Functional homogeneity of communities

We now assess how many communities are judged functionally homogeneous, looking in particular at how our results vary with resolution parameter.

Figures 2i) illustrate the number of communities judged to be functionally homogeneous, and Figures 2ii) show the number of proteins in communities judged to be functionally homogeneous, for a) the *A *network and b) the *P *network. We find that the large communities present at small values of the resolution parameter *λ* are not judged to be functionally homogeneous. As *λ* is increased, larger numbers of proteins occur in functionally homogeneous communities, peaking in the range 1.5 < log(*λ*) < 2. At log(*λ*) = 1.5, the mean community size is 73 proteins, and the majority of proteins, 3071 of 4980, are in functionally homogeneous communities as judged by our GO similarity measure. The shapes of the curves of both Figure 2a) and 2b) for all three similarity measures are very similar. Indeed, we find that the overlap between the communities judged to be functionally homogeneous between any two of the three measures is high; for example, it is 70% between the GO and correlated growth rates measure over almost the entire range of the resolution parameter in both *A *and *P *networks (see Figure S2 in Additional File 1 for the complete data). Given that the correlated growth similarity measure represents a very different data type to the GO and MIPS annotations, this agreement gives us confidence in the similarity measure we use for GO and MIPS. As we use only the top level of the MIPS functional annotations, we capture less information than the GO measure, so it is unsurprising that fewer communities are found to be functionally homogeneous under this measure.

The *P *network shows a similar pattern to the *A *network. One difference is that communities start to be judged as functionally similar at a slightly lower resolution. This is most likely due to the different topological properties of the *P *network. That there are comparably many functionally homogeneous communities in the *P *network as the *A *network is of interest, as communities found in *P *networks are found to be poor choices for predicting function on the basis of enrichment of terms [31].

For almost all proteins, there is some value of the resolution parameter that assigns them to a functionally homogeneous community. In fact 4652 out of 4980 *A *proteins and 5647 and of 5669 *P *proteins are in such communities at some value of the resolution parameter. For a given protein, it may not be that it interacts most closely with proteins involved in the same process. Indeed it is often necessary to look at a larger scale, placing the community in a bigger community in order to identify the biological processes it participates in. Whether or not this is the case, and which network scale (resolution) is most indicative of the processes a protein is involved in, will depend on the particular protein one is interested in. This demonstrates the biological motivation for investigating community structure at multiple resolutions, and suggests the desirability of a method to easily identify those communities most likely to be functionally homogeneous.

**Figure 3:**
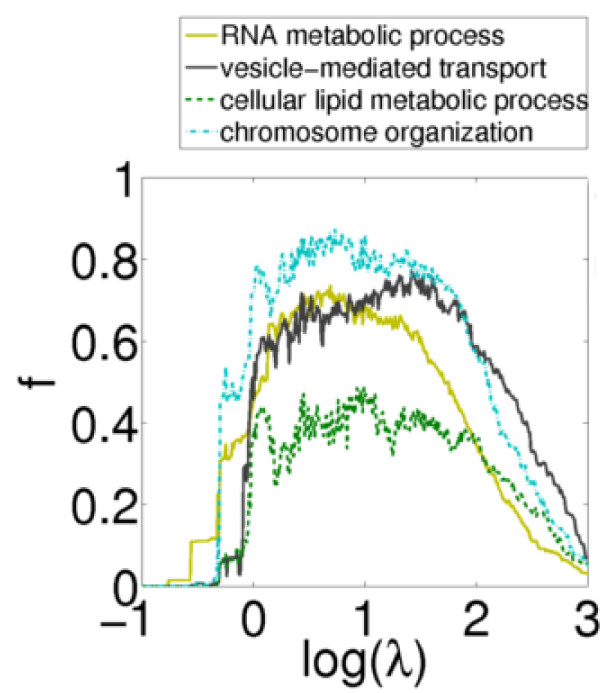
**Fraction of proteins of particular types in functionally homogeneous communities**. The fraction of proteins, *f*, of particular types that are in functionally homogeneous communities in the *A *network, with changing resolution parameter. With changing resolution parameter proteins of particular types have consistent differences as to how often they are found in functionally homogeneous communities. For example, proteins involved in chromosome organisation are far more likely to be in functionally homogeneous communities than proteins involved in metabolism. There are also some features that suggest 'good' resolutions for particular processes. For example, a good resolution for proteins involved in vesicular mediated transport would be log(*λ*) = 2.7 (for which the mean size of communities is 10), whereas for proteins involved in RNA metabolic processes, log(*λ*) = 0.8 would be better (the mean size of communities is 30).

We might expect proteins involved in particular processes to show different propensities to lie in functionally homogeneous communities. We focus on a set of general protein types (as defined and listed in the Methods), and investigate what fraction of each type of protein lie in communities judged functionally homogeneous under the GO measure through changing resolution parameter. Figure 3 illustrates for the *A *network these percentages for four particular processes. (Figure S3 in Additional File 1 shows the same figure for all 11 terms for the *A *network and separately for the *P *network). Proteins of some types are far more likely to be found in functionally homogeneous communities than others. For example, for both the *A *and *P *networks, proteins involved in chromosome organisation are far more likely to be found in functionally homogeneous communities than proteins involved in lipid metabolism. In addition, there are some indications that the resolutions of most interest can depend on the type of protein under investigation. As can be seen in Figure 3, proteins involved in RNA metabolic processes are more likely to be found in functionally homogeneous communities at log(*λ*) = 0.8, where the mean size of communities is 30. In contrast, proteins involved in vesicle-mediated transport are found in greater numbers in functionally homogeneous communities at log(*λ*) = 1.7, where the mean size of communities is 10.

### Examples of communities found at multiple resolutions

**Figure 4:**
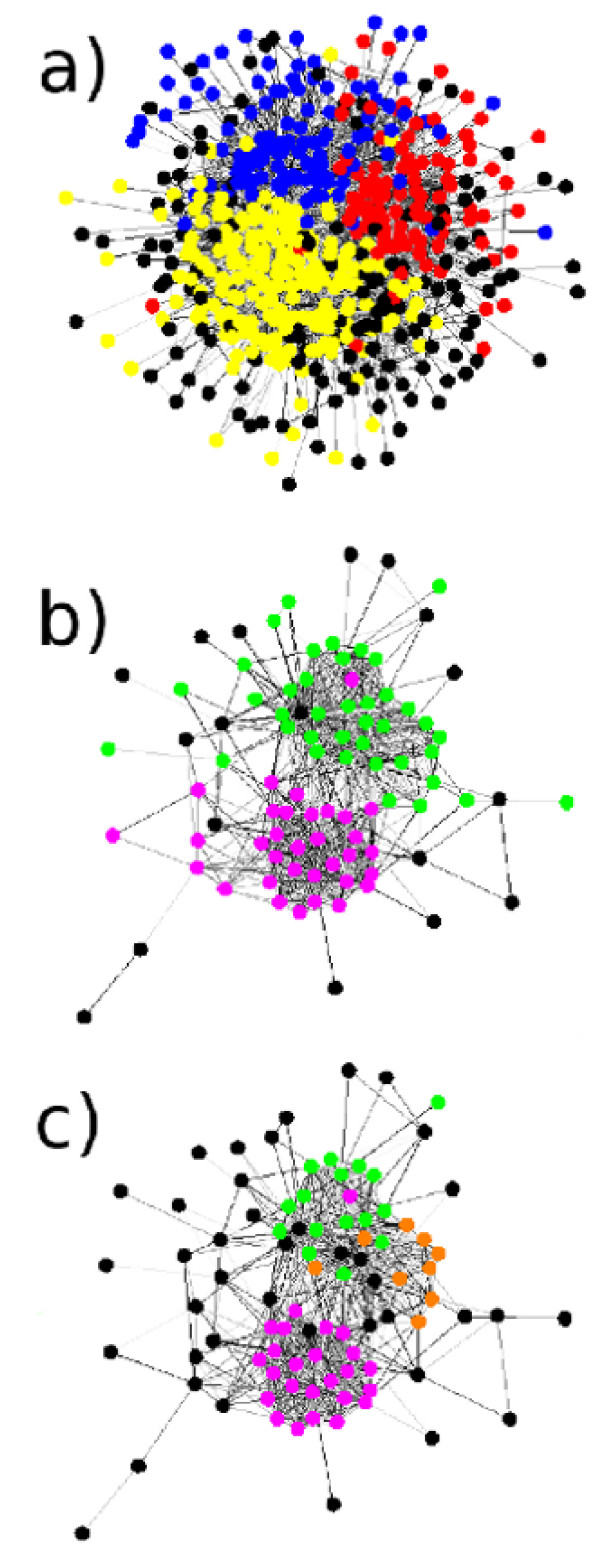
**Examples of communities found**. a) A representation of a community in the *A *network at resolution parameter value log(*λ*) = 0, with nodes (proteins) coloured according to the partition of this community at log(*λ*) = 0.5. The colours are the same as for Figure 1 a), where this group of proteins has labels roughly in the range 0 - 500. Almost all of the nodes have some relationship to the ribosome. The proteins in the yellow community are mostly ribosomal subunits, those in the red community are mostly pre-cursors to and processors of the small ribosomal subunit, and those in the blue community have similar roles to those in the red community but for the large subunit. The shading of the links has no significance; its purpose is to ease visualisation. Black nodes are not located in one of the three largest communities discussed in the text. b) A representation of a community at log(*λ*) = 0.5, with nodes (proteins) coloured according to the partition of this community at log(*λ*) = 0.75. The proteins identified at the lower resolution almost all play some role in transcription initiation. At the higher resolution, more structure is revealed: the pink community consists mostly of proteins from the RNA polymerase II mediator complex and the green community mostly consists of proteins from the TFIID and SAGA complexes. c) The partition at a higher resolution (log(*λ*) = 1.6). The green community from b) has split into the SAGA complex (green) and the TFIID complex (orange). The names and descriptions of the proteins in these example communities are given in Additional File 2. The node positions for visualisation were computed in the same way as for Figure 1.

Consider the community at log(*λ*) = 0 that is marked as the blue block in Figure 1 for the *A *network (over node labels approximately 0 to 500). This contains 528 proteins and consists largely of proteins with some relationship to the ribosome (based on short protein descriptions found on the SGD website). Figure 4a) shows this community, where we have coloured nodes according to the community partition at the later partition log(*λ*) = 0.5. The colours - red, yellow, and blue - are the same as in Figure 1, where most of the community present at log(*λ*) = 0 has split into three communities at log(*λ*) = 0.5. The blue community consists of 107 proteins, which are largely precursors to and processors of the large ribosomal unit. The red community consists of 95 proteins, which have a similar function but for the small ribosomal subunit. The yellow community has 190 proteins, 93 of which are constituents of the ribosome and the remainder of which are either of unknown function or associate to the ribosome. We give short descriptions of the proteins in these communities in Additional File 2.

An illustration of the biological relevance of community structure at three partitions is given in Figures 4b) and 4c). We show a community of 90 proteins at log(*λ*) = 0.5, and display its partition into communities at b) log(*λ*) = 0.75 and c) log(*λ*) = 1.6. Almost all of the proteins in the community at log(*λ*) = 0.5 play some role in transcription initiation. At log(*λ*) = 0.75 this community has split into two main smaller communities: the pink community contains constituent proteins of the RNA polymerase II mediator complex and the green community contains components of the closely related SAGA and TFIID complexes. At log(*λ*) = 1.6, this second community has split into the SAGA and TFIID complexes.

**Figure 5:**
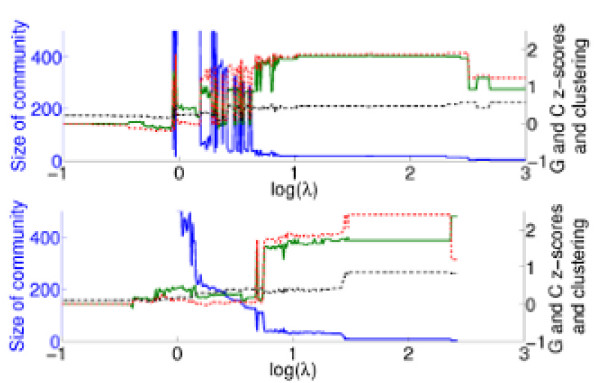
**Tracing the community membership of a particular protein through changing resolution**. For the example protein YCL008C, we show the size (solid blue curve), mean clustering coefficient (dot-dashed black curve), mean *z*-score under the GO measure (solid green curve), and correlated growth measure (dashed red curve) with changing resolution for the *A *network (top) and *P *network (bottom). Long plateaus in these properties represent robust communities. We give further examples in Additional File 1 Figure S4.

Multi-resolution community detection and characterisation is relevant both from the global viewpoint, where one can investigate the aggregate functional organisation of the proteome, and from the local perspective, where the community membership of particular proteins can be traced through changing resolution parameter. We thus now consider a protein-centred view of multi-resolution community detection. We consider, for an example protein, the properties of the communities to which it is assigned through changing resolution parameter, see Figure 5. The size of the communities, their mean similarity under the *G *and *C *measures, and the mean clustering coefficient are shown. The protein is a member of the ESCRT-I complex. (Figure S4 in Additional File 1 gives a further four examples.) Note the very robust properties of the communities in the *A *network over resolution parameter values of approximately 1 ≤ log(*λ*) ≤ 2.5, despite the tendency for them to be partitioned as *λ* increases. At these resolutions, the protein is in the same community as other members of the complex, as well as a few other very closely associated proteins. Beyond log(*λ*) = 2.5, the complex is broken up, as reflected in the drop in mean similarity values. The community present over 0.7 ≤ log(*λ*) ≤ 1.4 in the *P *network contains many proteins associated to the complex (in addition to the complex itself). Above the step observable at log(*λ*) = 1.4, only members of the complex are present. In Additional File 2, we give the names and brief functional descriptions of proteins that occur in some of the same communities for this example, and the four other examples given in Additional File 1. These five examples all show the following behaviour.

• In general, as would be expected, the size of the community to which a protein is assigned decreases with increasing resolution. There is often a large range of resolutions over which the community has constant size (which we have observed in practice to entail the same community across multiple resolutions). Such communities are particularly resilient to being split up at increasing resolutions, despite the tendency for them to be partitioned.

• The community similarity under the *G, C *and *M *measures often shows a close correlation.

• At higher resolutions, there tends to be a higher community similarity, as might be expected of a hierarchically organised system. This is, however, not always the case: community similarity can decrease at higher resolutions. In these instances, a group of proteins has been partitioned beyond the point at which function is shared, possibly through the exclusion of proteins involved in the same processes that do not necessarily directly interact with each other.

• There is often a large overlap between the community membership in the *A *and *P *networks, but it can also be quite different. For example, in Additional File 1 Figure S4 c), the protein occurs with other proteins in the same complex in the *A *network, whereas in the *P *network it occurs with non-complex members which are nonetheless involved in the same process. The functional homogeneity of communities can also be different: sometimes the protein occurs in many functionally homogeneous communities in the *A *network and not the *P*, and sometimes vice versa. This is unsurprising given the very different nature of *A *and *P *interactions. By treating them separately, we are able to pick out both types of pattern.

### Use of topological properties to select functionally homogeneous communities

Almost all proteins are in functionally homogeneous communities at some value of the resolution parameter, and we therefore devise a method to swiftly identify these resolutions, especially if there is a dearth of functional information. We investigate whether any easily-calculated topological properties of the communities can act as indicators of functional homogeneity. Given a protein of interest we can then use such measures to quickly identify 'good' resolutions, without the need to assess functional homogeneity.

We tested 26 topological properties for their ability to predict functional homogeneity using the AUC metric (see Methods), and show our results in Table 4. In general, the AUCs for the *P *network are lower than those for the *A *network, perhaps because there is more potentially usable information in the *A *network as it is significantly denser (see Table 1).

**Table 4:** Topological metrics tested and AUCs

	*A*			*P*		
Network topology measure	*G*	*C*	*M*	*G*	*C*	*M*
Mean degree	0.6476	0.6476	0.6142	0.5130	0.5373	0.5387
Degree assortativity coefficient [58]	0.6913	0.6913	0.6277	0.4799	0.5517	0.5181
**Clustering coefficient **[59]	**0.7186**	**0.7186**	**0.6613**	**0.5521**	**0.5829**	**0.5725**
Global mean Soffer clustering coefficient [60]	0.4857	0.4857	0.4819	0.3915	0.4735	0.4461
Local mean Soffer clustering coefficient [60]	0.4784	0.4784	0.4662	0.3892	0.4654	0.4540
Mean geodesic node betweenness centrality [61]	0.4600	0.4600	0.4973	0.5045	0.5094	0.4959
Mean closeness centrality [61]	0.5275	0.5275	0.5524	0.4877	0.4919	0.4815
Mean eigenvector centrality [61]	0.5601	0.5601	0.5722	0.5312	0.5551	0.5246
Mean information centrality [61]	0.5191	0.5191	0.5429	0.5253	0.5456	0.5170
Mean geodesic distance [59]	0.3839	0.3839	0.3717	0.4274	0.4945	0.5066
Diameter [61]	0.4457	0.4457	0.4042	0.4366	0.5004	0.5079
Mean harmonic geodesic distance [59]	0.4088	0.4088	0.4042	0.5024	0.4834	0.4995
Energy [59]	0.5237	0.5237	0.4982	0.4568	0.4976	0.5114
Entropy [59]	0.5655	0.5655	0.5327	0.5077	0.5127	0.5280
Off-diagonal complexity [62]	0.5941	0.5941	0.5457	0.5081	0.5054	0.5237
Cyclomatic number [62]	0.6331	0.6331	0.5733	0.5173	0.5300	0.5425
Connectivity [62]	0.6437	0.6437	0.5766	0.5245	0.5334	0.5468
Number of spanning trees [62]	0.4525	0.4525	0.4531	0.4451	0.4516	0.4491
Medium articulation [62]	0.5659	0.5659	0.4463	0.5295	0.5070	0.5592
Efficiency complexity [62]	0.5316	0.5316	0.5343	0.4911	0.4945	0.4982
Graph index complexity [62]	0.6564	0.6564	0.6492	0.5211	0.5469	0.5250
Density	0.6541	0.6541	0.6553	0.5277	0.5676	0.5235
Efficiency [63]	0.5790	0.5790	0.5896	0.4964	0.5071	0.4865
Fraction of articulation vertices [64]	0.5065	0.5065	0.5028	0.5216	0.5062	0.5091
Largest eigenvalue	0.6054	0.6054	0.5663	0.4941	0.5041	0.5185
Rich club coefficient [65]	0.5428	0.5428	0.5896	0.4988	0.5209	0.4868

The network topology measures tested and their associated AUCs. We report the results for using each of these as a predictor for functional homogeneity as judged under the three measures of functional similarity (GO, *G*, correlated growth rates, *C*, and MIPS, *M*) for both the *A *and *P *networks. The AUCs are given as the average performance over the range 0 ≤ log(*λ*) ≤ 3. The clustering coefficient (definition given in the text, equation 9) is the best predictor in all cases. (The topological properties were computed from code developed by Gabriel Villar.)

**Figure 6:**
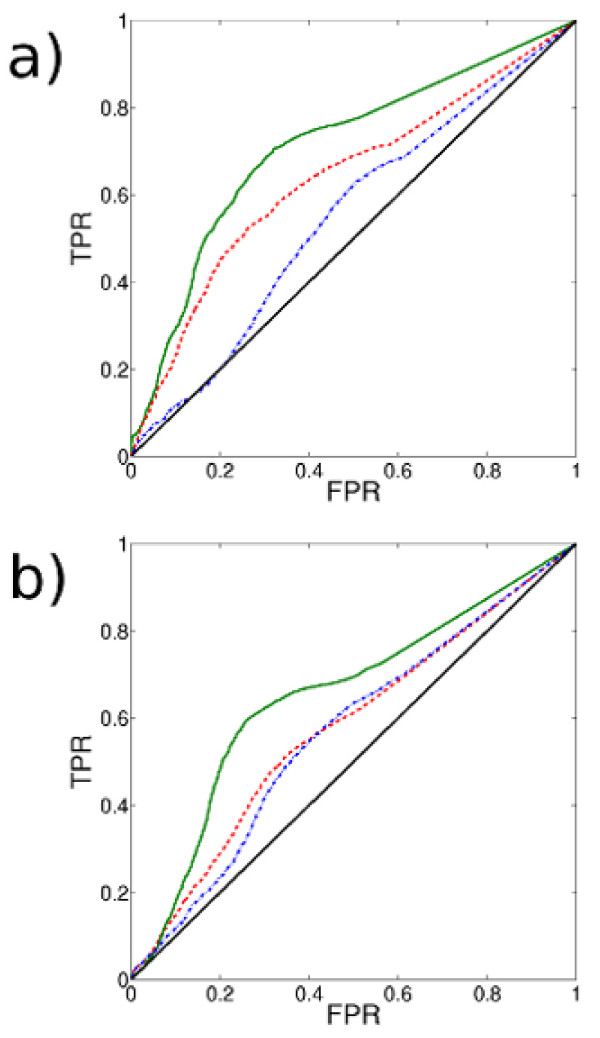
**ROC curves for using mean clustering coefficient to pick out functionally homogeneous communities in a) the *A *network and b) the *P *network**. The Receiver Operating Characteristic (ROC) curve for using mean clustering coefficient as a predictor of functional homogeneity under the GO measure (solid green curve), MIPS measure (dot-dashed blue curve) and correlated growth measure (dashed red curve). We plot the false positive rate (FPR) versus the true positive rate (TPR). A random classifier would give the solid black line. For the *A *network under the GO measure, a true positive rate of 70% is achievable with a false positive rate of 30%. For both networks, the best predictive ability is achieved for the GO measure, and the worst for the MIPS measure (see Table 4 for areas under the curves (AUCs).). The AUCs for the *P *network are in general lower than those for the *A *network (see Table 4).

We find that the clustering coefficient is the most useful of the topological properties tested in the prediction of functional homogeneity for all three similarity measures and in both the *A *and *P *networks. The clustering coefficient of a network is a measure of the mean local clustering around nodes: A node has a high clustering coefficient, *c*, if its neighbours are also neighbours of each other [54,55]. It is defined for each node as(9)

where *N*_triangle _is the number of triangles of which the node is a member, and *N*_triple _is the number of connected triples of which the node is a member. (A connected triple is a single node with edges running to an unordered pair of other nodes.) Figure 6 shows for a) the *A *network and b) the *P *network the ROC curves for using the mean clustering coefficient of nodes in a community as a predictor of functional homogeneity for each of the three similarity measures in the *A *network. (See Methods for a description of the construction.)

There is some element of discretion for annotating *A *type interactions, i.e. deciding which pairs to list interactions between following experiments, with the principle competing models referred to as 'matrix' and 'spoke' [56]. This choice could cause artefactual topological features, so the extent to which we find particular topological features correlating with functional homogeneity could be sensitive to annotation choice. We are therefore encouraged that the same trends in predictive ability are evident in the *P *network, for which there is no such element of discretion.

As can be seen from Figure 5 and the figures in Additional File 1 Figure S4, clustering appears to be a good proxy for functional homogeneity when looking at individual proteins, and in the absence of much functional information could guide which resolution(s) should be targeted for investigation.

## Conclusions

If protein interaction networks are to aid understanding of how biological function emerges from the concerted action of many proteins, then it is crucial to explore connections between network structure and biological function. In this paper we investigate how the function of sets of proteins varies with network community structure of yeast at multiple resolutions.

We find that community structure does indeed help identify sets of proteins that act together, and that this connection between network structure and biological function depends on what network scales are probed. We do not expect there to be any single scale of interest in this middle-scale structure of the protein interaction network; although previous studies have applied community detection algorithms to protein interaction networks, no study to our knowledge has investigated this structure at multiple resolutions. We find that 4652 of 4980 proteins in the *A *network, and 5647 of 5669 proteins in the *P *network, are in functionally homogeneous communities at some value of the resolution parameter as judged under the GO similarity measure. The number of proteins in functionally homogeneous communities peaks at about *λ* = 3 for the *A *network (which is beyond the standard 'modularity' resolution of *λ* = 1). For the *P *network the peak is less pronounced, with the actual maximum occurring at *λ* = 7 (i.e. log(*λ*) = 0.86). These findings emphasise that there are different scales of interest in the community structure of protein interaction networks, and that the one of primary interest will depend on which proteins and processes one is investigating. For some protein types, there are natural resolutions, at which more proteins of that type are assigned to functionally homogeneous communities. We also find that proteins involved in some processes are much more likely to be in functionally homogeneous communities than others. For example we find for both networks and across a range of resolutions that approximately 70 - 80% of proteins involved in chromosome organisation compared to 40% involved in lipid metabolism are in functionally homogeneous communities.

Having a good measure of functional homogeneity is central for our analysis. We approach this issue by using three different characterisations of functional similarity: two based on the GO and MIPS structured vocabularies respectively and one based on the growth rates of gene knock-out strains under different chemical conditions [32] (an independent and objective characterization of biological function). The prevalent method in the literature for assessing functional homogeneity of a group of proteins is inappropriate for communities, as the number of interacting pairs in a group must be taken into consideration. By defining similarity at the pairwise level, we have developed a fair test of functional homogeneity through a comparison of interacting pairs. We also capture the aggregate functional similarity of two proteins, overcoming the need to assess functional homogeneity on a term by term basis (although this is, of course, also possible once communities of particular interest have been identified). Our tests of functional homogeneity (which are not statistical tests in the conventional sense because of our desire to exclude the effects of sample size) using the three measures of similarity show a high level of agreement with each other, giving us confidence in our chosen measures of functional similarity.

Throughout this study, we have investigated two separate yeast protein interaction networks: that based on associations (the *A *network; mostly TAP-like data), and that based on physical associations (the *P *network; mostly yeast-two-hybrid data). We find that the two networks have similar properties with respect to their community structure, despite their very different global topological properties. Rather than regarding the yeast-two-hybrid data as of an inferior quality [31], we start from the basis that it is of a fundamentally different type and should thus be treated separately. We find similar percentages of functionally homogeneous communities in both networks.

As we have found a connection between network communities and biological function, we can use observed community structure to predict aspects of biological function. We find in particular that communities with a high mean clustering coefficient are far more likely to be functionally homogeneous than those with a lower one. The mean clustering coefficient of nodes within a community can therefore be used to predict that a group of proteins is functionally homogeneous, even in cases where our current knowledge does not allow us to infer this on the basis of functional annotations alone. These results give insights into the relationships between the structural and functional organisation of the cell considering the whole proteome.

We have also illustrated the utility of our framework for biologists who are interested in a particular protein. In a chosen interaction network, one can determine the community membership of the protein of interest at multiple resolutions. Even in the dearth of functional information, the easily-calculated clustering coefficient can be computed to suggest resolutions of particular interest.

In conclusion, we have linked the community structure of a protein interaction network with biological function by probing different scales of network structure. The identified communities are candidates for biological modules within the cell. We have also illustrated how this connection can be used to select groups of proteins that likely participate in similar biological functions.

## Authors' contributions

All four authors conceived of the study, and ACFL carried it out. All authors read and approved the final manuscript.

## Supplementary Material

Additional file 1 **Supplementary figures 1-4**. Click here for file

Additional file 2 **Tables of proteins in communities given in the Examples Section**. Click here for file
