# Supplementary material for: The function of communities in protein interaction networks at multiple scales
Source: BMC Syst Biol. 2010 Jul 22;4:100. doi: 10.1186/1752-0509-4-100 (PMC2917431; doi:10.1186/1752-0509-4-100)
Supplement: Additional file 1 — Supplementary figures 1-4. [file 1752-0509-4-100-S1.PDF]

Additional File 1 for 'The Function of Communities in Protein Interaction Networks at multiple scales'

Anna C F Lewis, Nick S Jones , Mason A Porter and Charlotte M Deane*

Email: Anna C F Lewis - lewis@stats.ox.ac.uk; Nick S Jones - nick.jones@physics.ox.ac.uk; Mason A Porter - porterm@maths.ox.ac.uk; Charlotte M Deane - deane@stats.ox.ac.uk;

*Corresponding author

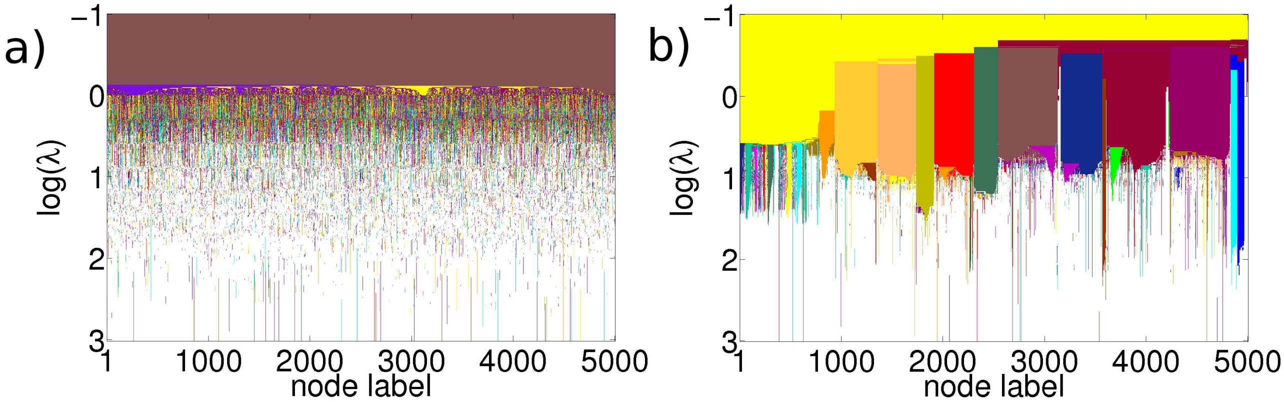

Figure 1: **As for Figure 1, but for a) An Erdös-Rényi random network and b) a network with strong community structure.** Both networks were designed to be of approximately the same size as the A and P networks (5000 nodes). The probability that two nodes are connected in the random network is the same as for the A network. We generated the network with community structure from code available at http://sites.google.com/site/santofortunato/inthepress2 (which is reported in Lancichinetti A, Fortunato S, Radicchi F: **Benchmark graphs for testing community detection algorithms**. *Physical Review E* 2008,**78**(4):46110). The parameters that we chose matched the statistics of the A network (average degree of 19, maximum degree of 1182), with additional parameters chosen as suggested default values (the exponent for the degree distribution is 2, the exponent for the community size distribution is 1, and the mixing parameter is 0.2).

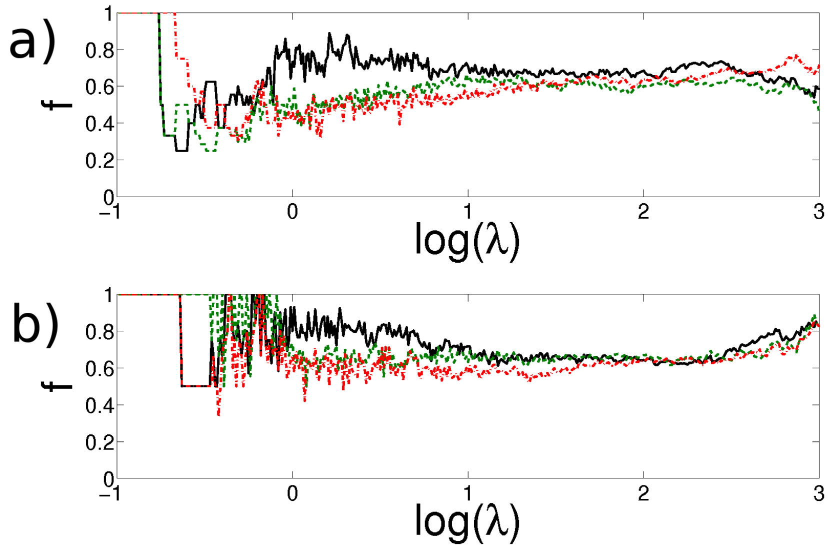

Figure 2: **The agreement in assessment of functional homogeneity between pairs of similarity measures.** For a) the A network and b) the P network, the fraction, f, of communities that are either both judged as functionally homogeneous or both not judged as functionally homogeneous under the G and C measures (black curve), the G and M measures (dark green dashed curve), and the M and C measures (red dot-dashed curve). The large degree of overlap between the measures derived from ontologies (G and M) with the measure derived from a single large scale experiment (C) gives us confidence in our ontology derived measures.

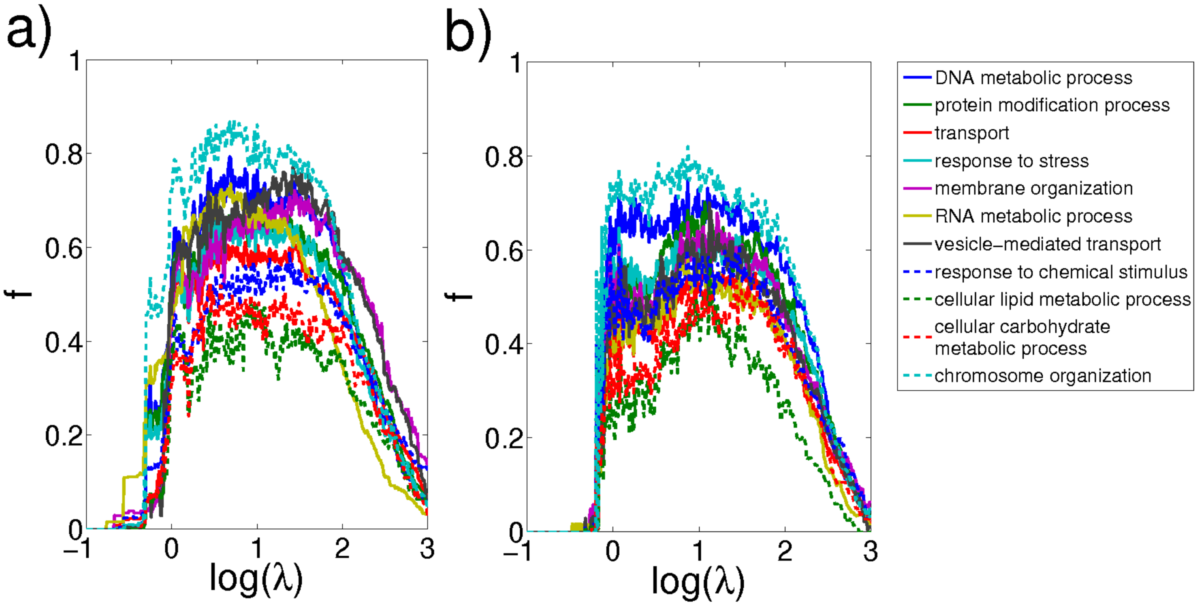

Figure 3: **The fraction of proteins of different types in functionally homogeneous communities as judged under the GO similarity measure.** The fraction, f, of proteins of particular types that are in functionally homogeneous communities in a) the A network and b) the P network, with changing resolution parameter. Some protein types are consistently more likely to be found in functionally homogeneous communities through changing resolution parameter. For example, proteins involved in chromosome organisation are much more likely to be in functionally homogeneous communities than proteins involved in metabolism. There are also some features that suggest 'good' resolutions for particular processes. The same patterns as for the A network hold for which types of protein tend to be classified in functionally homogeneous communities (see main text), but there do not appear to be any clear differences between protein types at varying resolutions in the P network, though some types have clearer peaks than others.

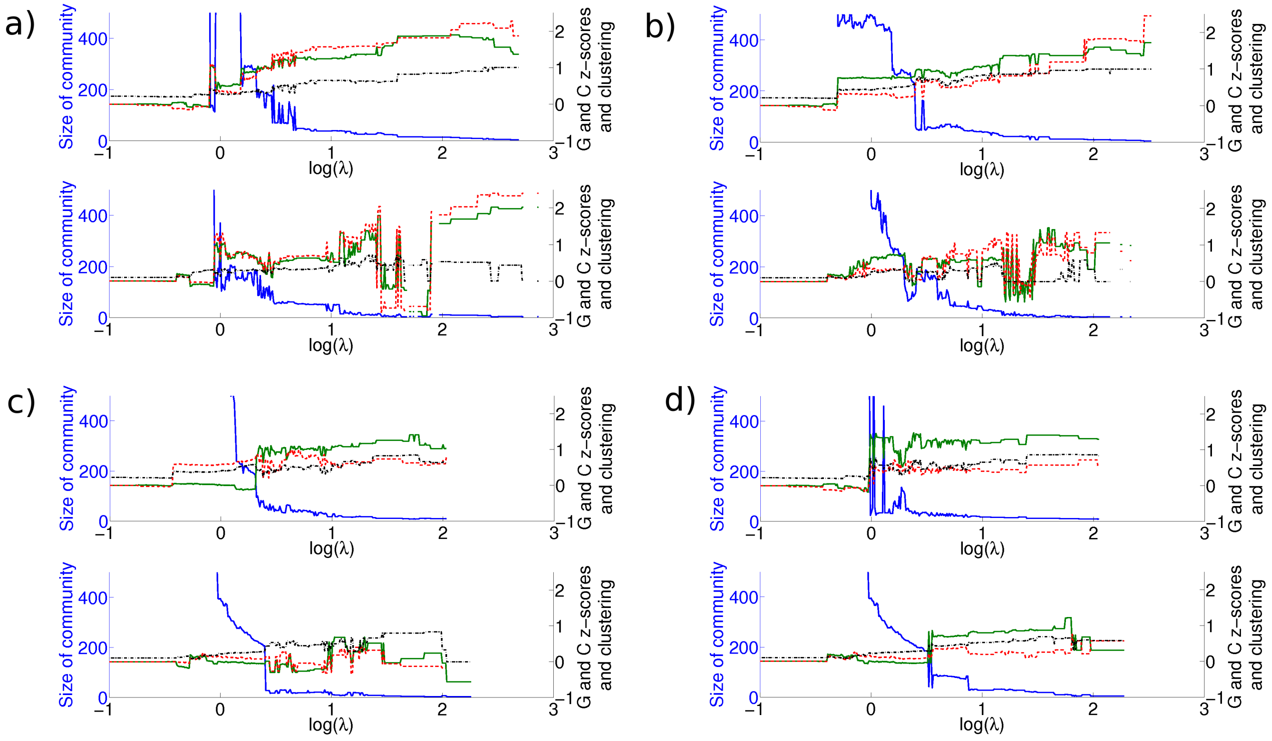

Figure 4: **Further examples as per Figure 5.** These figures display the same information as Figure 5, but for the proteins a) YAL002W, b) YAL011W, c) YAL016W, and d) YAL021C. We show the size (solid blue curve), mean clustering coefficient (dot-dashed black curve), mean z-score under the GO measure (solid green curve), and correlated growth measure (dashed red curve) with changing resolution for the A network (top) and P network (bottom). Gaps appear whenever the protein is assigned to a community of size three proteins or less. We give the names of proteins in several example communities, chosen as motivated by these figures, in Additional File 2.
